# Academia and society should join forces to make anti‐cancer treatments more affordable

**DOI:** 10.1002/1878-0261.13651

**Published:** 2024-04-18

**Authors:** Anton Berns

**Affiliations:** ^1^ Division of Molecular Genetics The Netherlands Cancer Institute Amsterdam The Netherlands

**Keywords:** anti‐cancer agents, clinical trials, comprehensive cancer centre, cost‐effectiveness, drug pricing, funding

## Abstract

Discovery research is the starting point for the development of more effective anti‐cancer treatments. It requires an interdisciplinary research environment with first‐class infrastructural support in which curiosity‐driven research can lead to new concepts for treating cancer. Translating such research findings to clinical practice requires complementary skills and infrastructures, including high‐quality clinical facilities, access to patient cohorts and participation of pharma. This complex ecosystem has yielded many new but also “me too” treatment regimens, especially in immuno‐oncology resulting in an extremely high pricing of anti‐cancer agents. The costs of antibodies, vaccines, and cell therapies charged by pharma stand out although the concepts and methodologies have been largely developed in academia, financed from public funds. Comprehensive Cancer Centres (CCCs) covering a coherent stretch of the cancer research continuum are well‐positioned to make these personalized treatments more affordable, but this will require restructuring of the way the translational cancer research continuum is funded.

## Graphical abstract

There is an urgent need to reconsider the way we organize drug development and assess their value for cancer patients and their cost‐effectiveness. Networks of comprehensive cancer centers and society at large need to take initiatives to play a more prominent role in the development of new anti‐cancer treatments.

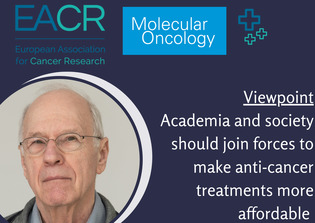

**Abbreviations**
CCC: Comprehensive Cancer Centre
CCCoE: comprehensive cancer centre of Excellence
EACS: European Academy of Cancer Sciences
EMA: European Medicines Agency
EMBL: European Molecular Biology Laboratory
EORTC: European Organisation for Research and Treatment of Cancer
ERC: European Research Council
FDA: U.S. Food and Drug Administration
JA CraNE: Joint Action EU Network Comprehensive Cancer Centres
OECI: Organisation of European Cancer Institutes

## 1 Introduction

Since cancer is an (epi)genetic disease mostly caused by the accumulation of mutations and genomic rearrangements, treating cancer is in fact fighting Darwinian evolution. This poses a major challenge for the development of effective systemic treatments, as resistance to therapy can easily arise. As a result, studies illustrating the effective killing of tumor cells *in vitro*, or in experimental animal models, does not guarantee successful treatment in patients with much larger tumor masses, often encompassing unique genomic aberrations, and specific microenvironments that can greatly modulate therapy response. Close interaction between preclinical and clinical researchers is therefore of great importance for the development of more effective therapies.

## 2 Comprehensive Cancer Centres of Excellence as the cornerstone

Comprehensive Cancer Centres of Excellence that combine high‐quality basic research with the capacity to clinically explore innovative concepts, facilitating bench‐to‐bed and bed‐to‐bench research, seem best equipped for bringing new promising laboratory findings to the clinic. Detailed understanding of the underlying biology and execution of the preclinical evaluation facilitates the design of informative clinical trials once suitable therapeutics become available. This also holds true for prevention and early detection research. Over the years, using Europe as paradigm, the European Academy of Cancer Sciences (EACS) has proposed strategies on how to make translational cancer research more effective, and high‐quality cancer therapeutics, cancer care and prevention more accessible and affordable for all European citizens. Comprehensive Cancer Centres (CCCs) and networks thereof are instrumental to achieve this [1].

However, although the importance of high‐quality CCCs as nuclei for advancing cancer treatments is now widely recognized [2], a comprehensive strategy to establish a sufficient number of such centers throughout Europe is still lacking. The Organization of European Cancer Institute (OECI) has established a valuable accreditation system for the comprehensiveness of cancer centers assessing multidisciplinary cancer research and cancer care criteria and is promoting the establishment of over a hundred such centers in Europe [3]. The EU program Joint Action Network of Comprehensive Cancer Centres (JA CraNA) has similar aims. However, for the development and testing of cutting‐edge new treatment paradigms additional qualities and advanced infrastructural provisions are needed. For this a modest number of CCCs with evident excellence (Comprehensive Cancer Centres of Excellence, CCCoE) covering a significant part of the cancer research continuum is indispensable [4]. Early on, the EACS has stressed the importance of such centers and has developed criteria, the Excellence Designation System, to permit their assessment [5].

**Fig. 1:**
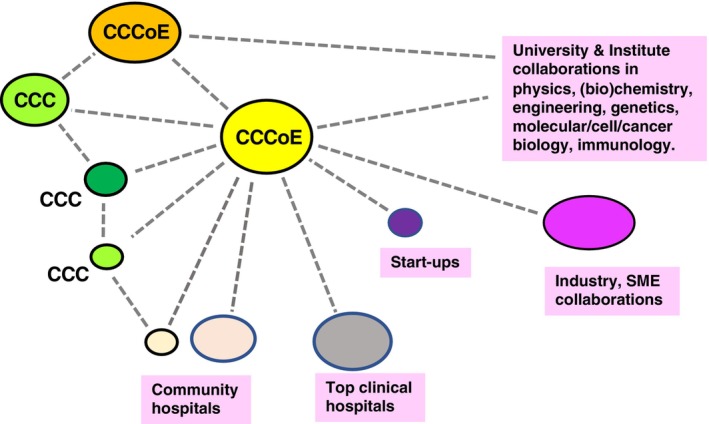
Network of collaborating research and clinical entities with a key role for Comprehensive Cancer Centers of Excellence (CCCoE). An interactive network of a Comprehensive Cancer Centre of Excellence (CCCoE) with a variety of similar centers and other partners together constituting an ecosystem in which all required expertise is available including the access to adequate patient cohorts to design and test innovative anti‐cancer therapies. CCC, comprehensive cancer center; SME, small and medium‐sized enterprises.

One of the primary tasks of a CCCoE is to develop new concepts and translate those into practice‐changing treatments to expedite the transition through the cancer research continuum including pivotal biomarker guided clinical trials. Close collaboration with other centers and hospitals (see Fig. 1) is needed to guarantee swift recruitment of patients and ensure that associated diagnostic tests [6] and patient monitoring meet the standards required for robust, data‐rich clinical trials that also can provide insight into benefit/cost relationship [7]. Such CCCs and networks thereof are not only well‐positioned to develop new treatments. They can also share responsibility, for example with overarching clinical trial organizations, such as the EORTC, to assess the value of new treatments in comparison with the standard of care in real‐world clinical practice and articulate their specific health benefits and cost‐effectiveness.

## 3 A prominent role for CCCoE in overseeing clinical trials

Currently, almost all the costs associated with late‐phase drug development and clinical trials are absorbed by the pharmaceutical industry. As a result, pharma determines the trial portfolio and largely dictates how trials are conducted, with concomitant dependencies on both hospitals and the clinicians running the trials [8]. Since academia is hardly a match in the negotiations with big pharma, this results in many “me too” trials. This not only impairs exploration of new treatment concepts, but in the absence of a proper functioning market (competition), it also leads ‐and has led‐ to an astronomical increase in the price of anti‐cancer medicines due to excessive profit margins [9]. On the contrary, the health benefits for patients and cost‐effectiveness of medicines is often modest or even non‐existent, as compared to standard of care [10].

To tackle this, a more distinctive fraction of new treatment paradigms than is the case now should be financed from public funds. Especially, personalized vaccines and cell‐ and gene therapies that are largely developed within academic settings, can be offered at substantially reduced costs when the property rights remain with academia and costs of clinical trials and registration are paid for by society [11]. Currently, roughly 80% of the cancer research budget is provided by pharmaceutical industry and therefore it is unrealistic to expect that this expense can be taken over by society, although it does pay for it indirectly by reimbursing the charges for the drugs. There is also no need for this. Rather it is important that this academic route becomes a realistic alternative (a decent competitor) for introducing new precision medicines into the market. This can also serve as a benchmark for what the reasonable costs are, that subsequently can be evaluated for their health benefits and cost‐effectiveness under real‐world circumstances [12]. We already witness some initiatives in this direction with academia‐controlled and society‐financed cell therapies and this route deserves further encouragement. Small biotech and pharma should be encouraged to participate but without sitting in the driver seat. Now, the system is broken, due to the way it incentivizes investigators, deficiencies in intellectual property legislation that is poorly tuned to medicine development, convoluted registration procedures, and the dominance granted to big pharma: “who pays the piper calls the tune” [10].

## 4 How can we establish a network of high‐quality centers?

Establishing CCCoEs throughout Europe is therefore a high priority. Incidental support from the European Union in the framework of the EU Cancer Plan and the Cancer Mission can incentivize this [2], but is insufficient to firmly establish an array of such centers. Long‐term, earmarked support is needed as part of the funding envelope of Member States to establish, benchmark and sustain such centers. Furthermore, a granting system should be put in place to facilitate the formation of networks of CCCoE, such as Cancer Core Europe [13], that can join forces to speedily execute these tasks [14]. To guarantee the sustainability of CCCoEs and networks thereof in the different countries, the founding of an intergovernmental organization should be considered to foster the establishment and sustainability of CCCs that meet high‐quality standards (akin to the EMBL model). This could be the “European Cancer Institute”. Such an organization could also facilitate the early translation trajectory towards clinical application, including PI‐initiated clinical trials, and support registration trials. In addition, it might act as umbrella for overarching services such as biobanks and data centers that provide curated sequence and anonymized patient records that can be accessed for research purposes.

## 5 How can we finance academically centered cancer therapy development?

If one considers the skyrocketing costs of many of the current anti‐cancer medicines and cell therapies and the many new products in the pipeline, it is evident that it will soon become unaffordable even for rich societies to pay for these treatments. A very modest fraction of the funds society is currently prepared to provide for these anti‐cancer medicines would already suffice to strengthen this complementary route of therapy development. These funds could be transferred to the above‐mentioned intergovernmental entity (European Cancer Institute). Such an institute with scientists and clinicians in charge could then be made responsible for the distribution of the funds in a transparent manner similar to what the ERC is successfully doing for basic research. Also, charities might want to chip in, and some actually already do. It would also require that market authorisation agencies (FDA/EMA) actively explore how they can facilitate academic registration procedures. A fantasy? Maybe, but it might be the only way to make anti‐cancer treatments affordable in the future and accessible for less privileged communities in Europe and in other parts of the world.

## Conflict of interest

The author declares no conflict of interest.
